# Cardiorespiratory health effects of gaseous ambient air pollution exposure in low and middle income countries: a systematic review and meta-analysis

**DOI:** 10.1186/s12940-018-0380-3

**Published:** 2018-04-18

**Authors:** Katherine Newell, Christiana Kartsonaki, Kin Bong Hubert Lam, Om Kurmi

**Affiliations:** 1 Clinical Trial Service Unit and Epidemiological Studies Unit, Nuffield Department of Population Health, University of Oxford, Oxford, UK; 2 Medical Research Council Population Health Research Unit (MRC PHRU), Nuffield Department of Population Health, University of Oxford, Oxford, UK

**Keywords:** Air pollution, LMICs, Meta-analysis, Systematic review, Cardiovascular, Respiratory

## Abstract

**Background:**

Lack of research on the effects of gaseous pollutants (nitrogen oxides [NO_x_], sulfur dioxide [SO_2_], carbon monoxide [CO] and ozone [O_3_]) in the ambient environment on health outcomes from within low and middle income countries (LMICs) is leading to reliance on results from studies performed within high income countries (HICs). This systematic review and meta-analysis examines the cardiorespiratory health effects of gaseous pollutants in LMICs exclusively.

**Methods:**

Systematic searching was carried out and estimates pooled by pollutant, lag and outcome, and presented as excess relative risk per 10 μg/m^3^ (NO_x_, SO_2_, O_3_) or 1 ppm (CO) increase pollutant. Sub-group analysis was performed examining estimates by specific outcomes, city and co-pollutant adjustment.

**Results:**

Sixty studies met the inclusion criteria, most (44) from the East Asia and Pacific region. A 10 μg/m^3^ increase in same day NOx was associated with 0.92% (95% CI: 0.44, 1.39), and 0.70% (0.01, 1.40) increases in cardiovascular and respiratory mortality respectively, same day NO_x_ was not associated with morbidity. Same day sulfur dioxide was associated with 0.73% (0.04, 1.42) and 0.50% (0.01, 1.00) increases in respiratory morbidity and in cardiovascular mortality respectively.

**Conclusions:**

Acute exposure to gaseous ambient air pollution (AAP) is associated with increases in morbidity and mortality in LMICs, with greatest associations observed for cardiorespiratory mortality.

**Electronic supplementary material:**

The online version of this article (10.1186/s12940-018-0380-3) contains supplementary material, which is available to authorized users.

## Background

A growing evidence base now highlights how both short (days) and long (years) term exposure to gaseous ambient air pollution (AAP) (mainly consisting of nitrogen oxides [NO_x_], sulfur dioxide [SO_2_], ozone [O_3_] and carbon monoxide [CO]) is associated with a range of cardiorespiratory health outcomes [1–5]. The relevant mechanisms are thought to include systemic inflammation [6, 7], oxidative stress [8] and altered cardiac autonomic function [9, 10]. AAP is now listed as the one of the greatest environmental threats to health, estimated to result in 2.9 million global deaths annually [11]. Its widespread distribution plus lack of an observable threshold below which no health impacts are thought to occur makes it a global public health concern of growing importance. Levels of gaseous AAP have increased rapidly in low and middle income countries (LMICs), resulting from accelerated economic growth and unplanned industrialization often at the sacrifice of adequate environmental controls, thus LMICs are now consistently experiencing the greater burden of gaseous AAP and over 85% of AAP-attributable deaths [12].

However, the corresponding evidence base remains largely unreflective of this growing burden in LMICs with most AAP research (particularly large multi-city studies) performed within high income countries (HICs), predominantly North America [13, 14] and Western Europe [5, 15]. Consequentially this lack of research on the health effects of gaseous AAP within LMICs means results from HICs are often extrapolated to LMICs. However, such extrapolation ignores intrinsic differences between LMIC and HIC pollutant sources, composition and spatial variability as well as the underlying population and healthcare characteristics. For example, major sources of AAP within HICs are mainly traffic and industry, in LMICs however major sources can include traffic and industry in addition to the burning of biomass and solid fuels. Therefore, before relying exclusively on evidence from HICs to demonstrate the health effects of gaseous AAP in LMICs it is first necessary to explore the evidence within LMICs, minimizing the spatial uncertainty introduced by including estimates from HICs. The aim of this systematic review and meta-analysis is to examine the cardiorespiratory health effects of gaseous AAP exposure for adults in LMICs exclusively.

## Methods

The review protocol was registered with PROSPERO a priori (registration CRD42016051733) and adhered to the Preferred Reporting Items of Systematic Reviews and Meta-Analysis guidelines [16].

### Search strategy

We systematically searched PubMed, Web of Science, Embase, LILACs, Global Health and ProQuest for studies up until the 28th November 2016 using the following keywords “air pollution”, “nitrogen dioxide”, “nitric oxide”, “sulfur dioxide”, “ozone”, “carbon monoxide” plus appropriate terms for cardiorespiratory outcomes and LMICs (for full search strategy see Appendix 1). Hand searching of the applicable literature was also performed in relevant journals and bibliographies of included studies.

### Eligibility

Studies were included if they examined the cardiorespiratory effects of gaseous AAP (NO_x_, SO_2_, O_3_ and CO) in adults and were performed within LMICs (as defined by the World Bank Classification [17]). All included studies examined cardiorespiratory (10th revision of the International Classification of Diseases [ICD10]: I00-I99/J00-J99) mortality and/or morbidity in adults (aged ≥18 years). Study duplicates were removed and abstracts screened independently by two authors (CK and KN), any disagreements were resolved via inclusion of a third investigator (OK). Final selected studies were selected based on the inclusion criteria that they have attempted to control for the main confounding variables (including season, and meteorological trends, plus smoking and existing health conditions for cohorts), feature recorded cardiovascular/respiratory health outcomes (deaths and hospital admissions/emergency room visits), and have both AAP and health outcomes recorded quantitatively. We placed no restrictions on study design however only studies published in the English language were included. Where additional data was required authors were contacted.

### Data extraction

Data were extracted by one author (KN) and recorded in an Access database. The following characteristics were extracted from each study; study design, study population demographics, study period, pollutant type, type of cardiorespiratory events, region, city, confounders addressed, exposure classification method, analysis methods and effect estimates by pollutant, outcome and associated lag time. 10% of extracted study data were reviewed by a second author (OK) with no disagreements found. For the remaining studies, the other three coauthors independently checked and verified the extracted data. To show the association between NO_x_, SO_2_ and O_3_ with cardiorespiratory mortality/morbidity, standardized effect estimates were calculated per 10 μg/m^3^ increase in pollutant, for CO 1 ppm was used as the standard increment. These were the standard metrics used in most studies, however when this was not the case estimates were converted using the formula (1) shown in Additional file 1.

### Risk of bias

Since there continues to be no standardized validated framework for assessing risk of bias in environmental epidemiological studies, risk of bias was assessed regarding the following biases determined a priori; detection bias, selection bias, exposure classification bias, and the confounders addressed. As many epidemiological studies often use fixed site monitoring as a surrogate for personal exposure we expected some degree of exposure classification bias in most included studies. For studies assigning exposure status with > 3 fixed site monitors we assigned moderate risk of exposure bias, while those that used ≤3 we assigned high risk. Studies that used atmospheric modelling or personal exposure measurements were assigned low risk (see Appendix 2 for full exposure classification bias assessment). High risk of exposure classification bias was also assigned if studies were performed prior to 1980 due to poorer methodological and technological accuracy in quantifying and assigning AAP exposure. Studies without clinically confirmed outcomes or ICD coding in the quantification of health outcomes were considered high risk of detection bias, while those without representative study populations were considered high risk of selection bias. Finally, studies that did not adjust for at least three of the main confounding variables including seasonality, long term trends, influenza, weather, and population characteristics and lifestyle factors also had high risk of bias assigned.

### Statistical analysis

Due to the expected heterogeneity from included study designs, locations, and pollutant/population characteristics it was anticipated one “true effect size” would be unlikely to be observed across studies, therefore estimates were pooled using the random effect model accounting for variation both within and between studies. Estimates were pooled by pollutant (NO_x_, SO_2_, O_3_, CO), outcome (cardiorespiratory mortality/morbidity) and associated lag time (in days), and presented as the percent excess relative risk per 10 μg/m^3^ or 1 ppm increase in pollutant at a significance level of 0.05. To ensure results for one region were not biased toward a single city, where duplicate studies were found examining the same city during the same study period one estimate only was included in meta-analysis. Where this duplication occurred, estimates were selected by the following criteria (1) multi-city studies were prioritized over single city studies due to their often-higher methodological rigor, (2) the study with the lower assigned risk of exposure assessment bias was selected.

Pre-specified subgroup analysis was performed (providing study numbers were sufficient [> 2]) examining estimates by specific cardiorespiratory outcomes, city, co-pollutant adjustment, and those with high risk of bias removed. As we expected the number of estimates available for subgroup analysis to markedly lower than that of the main analysis, estimates for sub-group analysis were therefore pooled for lags 0–3 inclusively providing enough estimates for meta-analysis. Publication bias was assessed via construction of funnel plots with trim and fill, and quantified using Egger's test. If high heterogeneity was present particularly after subgroup analysis meta-regression was performed exploring the likely sources of variation. All analysis was performed in R [18].

## Results

**Fig. 1:**
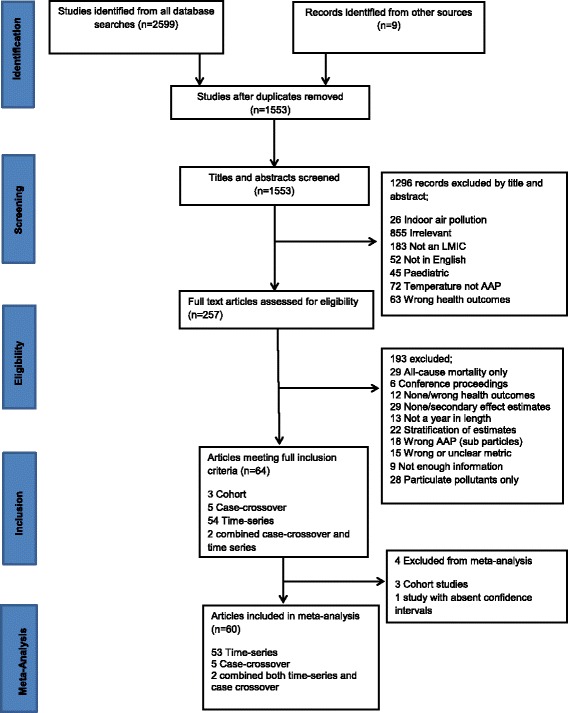
Study selection for the cardiorespiratory impacts of gaseous air pollution in LMICs

We reviewed the titles and abstracts of 1553 studies of which 64 met our pre-specified inclusion criteria (see Fig. 1). Prior to meta-analysis one study was excluded due to absence of confidence intervals, and an additional three cohort studies [19–21] examining long-term health effects of gaseous AAP exposure in China (representative of two cohort studies) were also excluded. This was due to insufficient number of estimates available for meta-analysis as well as the fact that they were all performed in China (two of which both in Shenyang). This left 60 studies for meta-analysis incorporating around 1.2 million events from eight countries examining acute health effects. Of the included studies 53 were time-series [4, 22–73], five case-crossover [74–78] and two incorporated both study designs [79, 80]. Most (44) were from East Asia and Pacific region only 11 were from Latin America and Caribbean and far fewer were included from Middle East and North Africa [2], Europe [2] and Africa [1] (see Additional file 1 for table of included study characteristics).

### Mortality

**Fig. 2:**
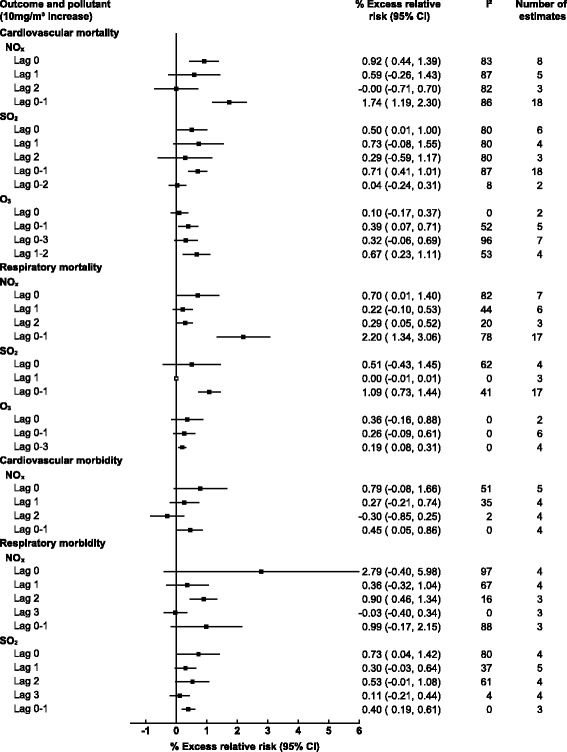
Pooled associations between gaseous ambient air pollution and cardiorespiratory mortality/morbidity stratified by outcome and lag time (days)

**Fig. 3:**
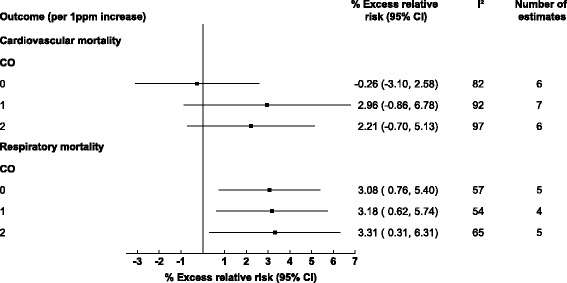
Pooled associations between carbon monoxide and cardiorespiratory mortality stratified by outcome and lag time (days)

Thirty-six studies examined the associations of gaseous AAP with mortality, seven of which focused on cardiovascular outcomes, four on respiratory outcomes and 25 included both. Of the included studies 30 included NO_x_, 25 SO_2_, 10 O_3_, and only five examined CO. A 10 μg/m^3^ increase in same day NO_x_, and SO_2_ was associated with 0.92% (95% CI: 0.44, 1.39) and 0.50% (0.01, 1) increases in cardiovascular mortality respectively, while no significant associations were observed for same day O_3_ or CO and cardiovascular mortality (see Figs. 2 and 3). For respiratory mortality and same day AAP only CO and NO_x_ produced associations, with 3.08% (0.76, 5.40) and 0.70% (0.01, 1.40) increases respectively. However, when using a moving average lag of 0–1 days NO_x_, and SO_2_ were both significantly associated with respiratory mortality, 2.20% (1.34, 3.06), and 1.09% (0.73, 1.44), respectively. The same trend was observed for cardiovascular mortality where a 0–1 moving average lag increased associations with a 1.74% (1.19, 2.30), 0.71% (0.41, 1.01), and 0.39% (0.07, 0.71) increased risk of cardiovascular mortality for NO_x_, SO_2_ and O_3_ respectively. The trend did not persist when the moving average lag was increased for SO_2_ to 0–2 days and for O_3_ to 0–3 days with no associations for cardiovascular mortality observed.

**Fig. 4:**
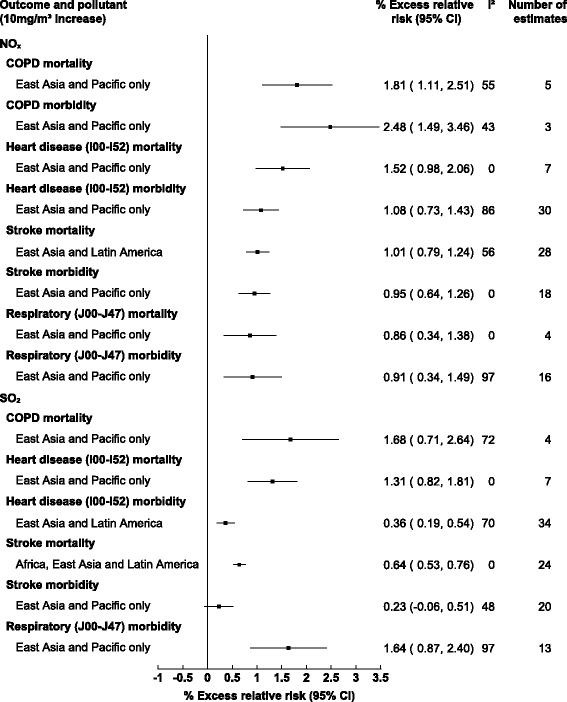
Pooled associations between gaseous pollutants and cardiorespiratory mortality/morbidity stratified by specific health outcomes (lags 0–3 days inclusively)

For mortality by cause specific outcomes the greatest associations were for NO_x_ and SO_2_ with chronic obstructive pulmonary disease (COPD), with a 1.81% (1.11, 2.51) and 1.68% (0.71, 2.64) increase in mortality within East Asia and Pacific exclusively (see Fig. 4). For stroke mortality, study results from Latin America and Caribbean results were pooled with those from East Asia and Pacific due to limited numbers of study estimates, however, the association remained significant with a 1.01% (0.79, 1.24) and 0.64% (0.53, 0.76) increase in stroke mortality for NO_x_ and SO_2_ respectively. No associations were observed for O_3_ and cause specific mortality, while too few estimates were available for CO.

### Morbidity

Twenty-four of the included studies examined gaseous AAP and morbidity of these 11 examined cardiovascular outcomes, five respiratory and seven included both. Eighteen studies included NO_x_, 20 SO_2_, five O_3_ and five CO. No associations were observed for gaseous AAP and cardiovascular morbidity apart from NO_x_ at a 0–1 day moving average with 0.45% (0.05, 0.86) increased risk. For respiratory morbidity, only SO_2_ was significantly associated with 0.73% (0.04 1.42) and 0.40% (0.19, 0.61) increases for same day and lag 0–1 respectively. Too few estimates were available to quantify the association of O_3_ and CO with morbidity.

For cause-specific morbidity the greatest associations were observed for NO_x_ which resulted in 2.48% (1.49, 3.46) increased COPD morbidity (see Fig. 4). NO_x_ was also associated with 0.95% (0.64, 1.26) and 1.08% (0.73, 1.43) increases in stroke and heart disease (I00-I52) morbidity respectively. For SO_2_ significant associations were only observed for respiratory morbidity (J00-J47) and heart disease morbidity (I00-I52) 1.64% (0.87, 2.40) and 0.36% (0.19, 0.54) respectively, however heterogeneity was high for both estimates. For respiratory morbidity the source of this heterogeneity could not be determined through meta-regression however for heart disease morbidity the pooling of lag times was found to be a significant source of the observed heterogeneity (*p* = 0.0057).

### Subgroup analysis

**Fig. 5:**
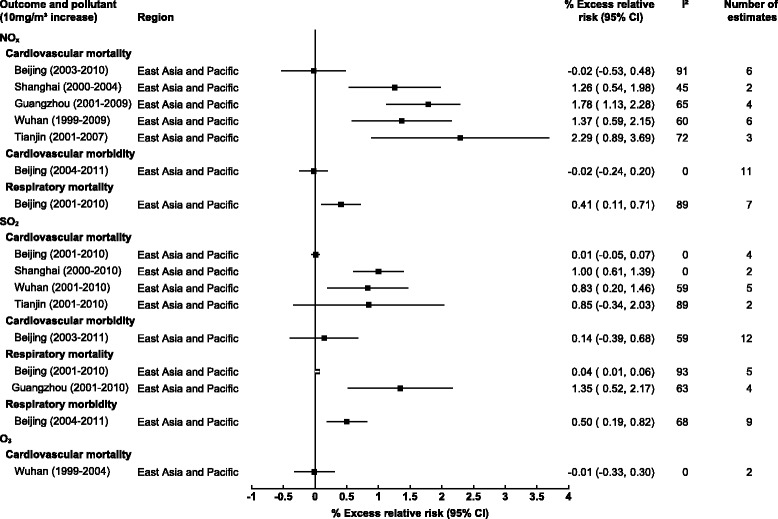
Pooled associations between gaseous pollutants and cardiorespiratory mortality/morbidity stratified by city (lags 0–3 days inclusively)

Due to the limited number of studies for regions other than East Asia and Pacific, estimates were unable to be pooled by pollutant and region. However, we were able stratify results by city for those within East Asia and Pacific region with large spatial variations apparent (see Fig. 5). For cardiovascular mortality, the greatest observations for NO_x_ and SO_2_ respectively were observed in Tianjin and Shanghai, with 2.29% (0.89, 3.69) and 1.00% (0.61, 1.39) increased cardiovascular mortality. For NO_x_ and respiratory mortality only Beijing provided enough estimates for meta-analysis with a 0.41% (011, 0.71) increase, however heterogeneity was high. For SO_2_ and respiratory mortality the observed association was greater in Guangzhou versus Beijing, 1.35% (0.52, 2.17) and 0.04% (0.01, 0.06) respectively, however again high heterogeneity persisted. The only significant association observed for SO_2_ and respiratory morbidity was in Beijing at 0.50% (0.19, 0.82).

**Fig. 6:**
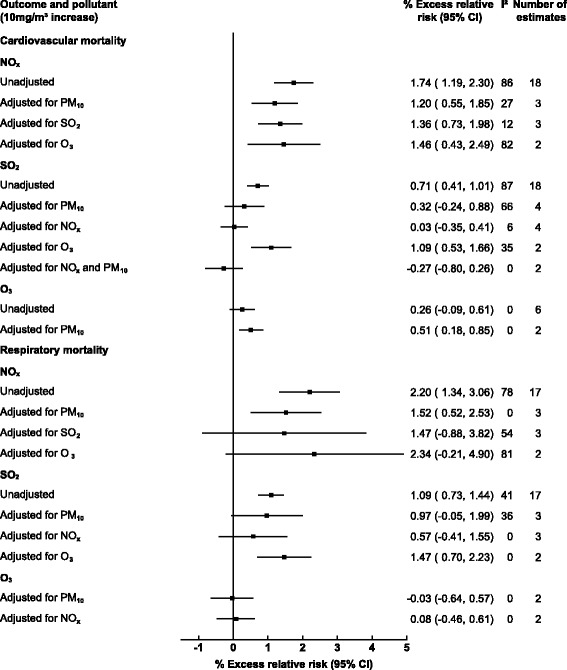
Pooled association between gaseous pollutants and cardiorespiratory outcomes adjusted for additional pollutants (lags 0–3 days inclusively)

Several studies provided estimates which allowed for stratification by co-pollutants at a moving average lag of 0–1 days. For NO_x_ and cardiovascular mortality, adjusting for PM_10_, SO_2_ and O_3_ attenuated the observed associations however all adjusted estimates remained statistically significant (Fig. 6). For NO_x_ and respiratory mortality adjusting for additional pollutants attenuated the associations, with adjustment for SO_2_ and O_3_ eliminating the observed associations entirely. When examining SO_2_ and cardiovascular mortality adjustment for PM_10_, NO_x_ and NO_x_ plus PM_10_ eliminated the observed association, while adjusting for O_3_ resulted in increased association from 0.71% (0.41, 1.01) to 1.09% (0.53, 1.66) although the number of available estimates were small. The same trend was observed for SO_2_ and respiratory mortality where adjustment for O_3_ increased the magnitude of the observed association from 1.09% (0.73, 1.44) to 1.47% (0.70, 2.23).

### Publication bias and heterogeneity

Publication bias was evident for NO_x_ and its association with both cardiovascular mortality (*p* < 0.0001) and respiratory morbidity (*p* = 0.0166), the same was observed for SO_2_ (*p* < 0.0001, *p* = 0.0022), however adjustment via trim and fill made minimal difference to the magnitude or direction of the observed associations (see Additional file 1). No publication bias was observed for any pollutants and cardiovascular morbidity, however SO_2_ and NO_x_ with respiratory morbidity did display evidence of publication bias. No publication bias was observed for O_3_ or CO although study numbers were small.

Many of the pooled estimates presented high heterogeneity following stratification these included estimates for NO_x_ and SO_2_ with both cardiovascular and respiratory mortality in Beijing and SO_2_ with cardiovascular mortality in Tianjin. Exploring this heterogeneity through meta-regression NO_x_ and cardiovascular mortality in Beijing the pooling of both study period and pollutant level were significant (*p* = 0.005, *p* = 0.004) sources of the observed heterogeneity. However, for NO_x_ and respiratory mortality in Beijing none of the extracted data variables were significant in explaining the between study variation with residual heterogeneity likely resulting from other variables not extracted, the same was observed for SO_2_ and respiratory mortality in Beijing with no sources of heterogeneity established through meta-regression. For SO_2_ and cardiovascular mortality in Tianjin there were too few studies to reliably examine heterogeneity through meta-regression.

**Fig. 7:**
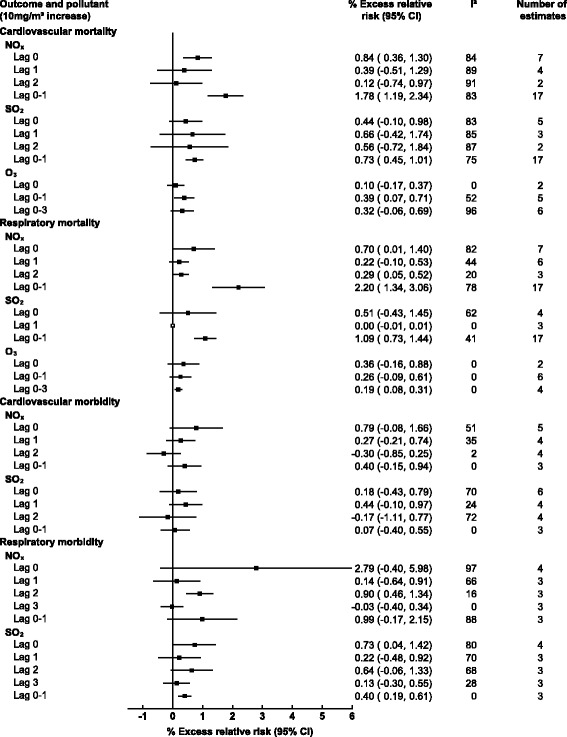
Pooled associations between gaseous pollutants and cardiorespiratory outcomes with studies with high risk of bias removed

In terms of bias, no studies were assigned high risk of selection or detection bias and all adjusted for at least three of the main confounding variables. However, 15 were assigned high or unclear risk of exposure classification bias. For cardiovascular mortality removal of these studies from meta-analysis tended to reduce the observed associations at shorter lags but increase estimates at longer lags NO_x_ and SO_2_ at a lag of 0–1 days increased from 1.74% to 1.78% and 0.71% to 0.73% respectively (Fig. 7). At shorter lags (lag 0) however the associations with cardiovascular mortality were attenuated from 0.92% to 0.84% and 0.50% to 0.44% for NO_x_ and SO_2_ respectively. The same trend was not observed for morbidity were removal of studies with high/unclear risk of bias produced more spurious alterations in associations and all estimates remained insignificant.

## Discussion

To our knowledge this is the first systematic review and meta-analysis to examine the effects of gaseous AAP in LMICs exclusively. Estimates were pooled from eight countries across five World Bank Regions the majority from within East Asia and Pacific (particularly China). The lack of studies from regions such as Sub-Saharan Africa and South Asia is likely not due to our English language restriction, but rather the lack of pollution monitoring stations in these regions making potential research unfeasible.

Comparing our results to a recent systematic review and meta-analysis conducted on the cardiorespiratory health effects of AAP globally [81] for certain gaseous pollutants we obtained similar results. For example, the greater associations we observed for SO_2_ and cardiorespiratory morbidity versus mortality was also seen in this study of the global association (2.33% [1.31, 2.87] and 0.70% [0.30, 2.10] for morbidity and mortality respectively). They found less discrepancy between NO_2_ cardiorespiratory mortality and morbidity (1.61 [1.31, 1.92] and 1.92 [1.41, 2.63] respectively) than observed in our results, however this may have been down to their pooling of cardiovascular and respiratory outcomes collectively versus our stratification of cardiovascular and respiratory events independently. As expected, similarly to us they also observed high spatial variation in the health effects of AAP.

Gaseous AAP was associated with both cardiorespiratory morbidity and mortality; however results were more consistent for mortality as well as for moving average measures of AAP as opposed to single day. This kind of temporal variability is consistent with evidence from both developed and developing countries [61] and potentially explained by the grouping of health outcomes with varying onset times.

Due to the limited number of studies from regions other than East Asia and Pacific stratifying estimates by region was not possible and therefore the variations in effects between LMIC regions were unable to be examined. However, we could examine differences in observed associations between LMIC (East Asia and Pacific) cities. The variation that we observed in associations between LMIC (East Asia and Pacific) cities is likely due to inherent differences in pollutant sources, composition and spatial variability that persist even within LMIC regions and countries. For example, differing spatial variability of pollutants is common even across relatively small areas due to geographical, topographical, and meteorological variation. Variability in pollutant sources are also commonly seen within LMIC countries, for example the increase in AAP in Northern China predominantly from abundant coal combustion used for heating in winter months [82]. However, the lack of association demonstrated in Beijing could have additional explanations. Consistently high levels of AAP in Beijing may have resulted in some degree of mortality displacement; additionally, Beijing’s healthcare infrastructure where migrants are not able to access healthcare within the city may be resulting in a misleading “healthier” population and null based bias introduced. Furthermore, the temporary reduction in AAP over the 2008 Beijing Olympic games may also explain the smaller associations observed with acute health outcomes, as studies have been pooled which include this period of reduced AAP.

As expected, our results for NO_x_ and mortality are like those obtained from the (albeit limited number) of multi-city studies examining the acute effects of gaseous AAP within LMIC regions [32, 61]. For example, a time-series study conducted for 17 cities in China [32] found NO_2_ at a 0–1 day was found to be associated with a 2.52% [1.44, 3.59] and 1.80% [1.00, 2.59] increase in respiratory and cardiovascular mortality respectively similar to our results (2.20% [1.34, 3.06]and 1.74% [1.19, 2.30] respectively). However, the majority of these studies examine mortality only and further multi-city studies incorporating morbidity are warranted. There are clear differences between our results and those performed within HICs exclusively, for example the APHEA-2 study of 30 European countries found smaller associations between NO_x_ and both cardiovascular and respiratory mortality at lag 0–1, 0.40% (0.29, 0.52) and 0.38% (0.17, 0.58) respectively [5]. A potential explanation could be the increasing NO_x_ emissions within LMICs resulting from rapid industrialization and motorization leading to substantial increases in NO_x_ concentrations [83]. Our results for O_3_ were however similar regarding cardiovascular mortality to those observed in HICs [1, 84]. The increase in effect estimates for O_3_ size across longer time lags observed in our study is also consistent with evidence from developed regions [85] likely due to a combination of pooling of health outcomes with varying onset times and potentially delayed inflammatory responses. Our results for CO are smaller than those observed in HICs [86] and potential explanations could include higher indoor CO levels and smoking rates in LMICs resulting in increased tolerance to outdoor CO increments. However, the studies incorporating CO are limited with more research required.

The differences in observed associations between gaseous AAP and cardiorespiratory health impacts in HICs versus LMICs is likely due to the differences previously stated including underlying pollutant sources, composition, spatial variability, in addition to population and healthcare characteristics. Pollutant sources in LMICs face less regulation than in HICs regarding location, magnitude, and chemical composition of emissions. For example, a lack of emission standard laws in many LMICs results in higher unregulated point source emissions, greater emissions from low quality gasoline and diesel, and emissions from pollutant sources not commonly found in HICs such as open burning. LMICs also face lower life expectancies in addition to poorer healthcare provision than HICs. Furthermore, while gaseous AAP such as NO_x_ has declined in many HIC countries in LMICs it is present at much higher levels.

Comparing our results to those from high income areas that lie within LMIC regions such as Hong Kong, our results are similar for both cardiovascular and respiratory mortality [61]. However associations from Hong Kong for gaseous pollutants with morbidity are greater than those observed here [87] possibly due to greater uptake and availability of healthcare in these HICs, rather than climatic or pollutant differences as these are likely to be similar to that of nearby LMIC China. For example, Hong Kong frequently endures similar AAP levels to those seen in southern China. Hong Kong AAP also has a similar composition and emission sources to that of China particularly with transboundary pollution such as windblown smog from the Guangdong area. In terms of regulation, Hong Kong like LMICs has air quality objectives rather than stringent standards seen in HICs such as those within European Union.

For cause specific outcomes, the greatest associations observed for COPD are consistent with the evidence base which includes observational studies from both developed [88] and developing [89] countries as well as toxicological studies [90]. NO_x_, SO_2_ and O_3_ are all acknowledged in their capacity to induce increased reactive oxygen species (ROS) production and oxidative stress resulting in localized pulmonary as well as systemic inflammation. NO_x_ and SO_2_ were also significantly associated with stroke with slightly stronger associations observed for mortality than morbidity, possibly due to fewer individuals surviving strokes in LMICs.

### Strengths and limitations

This is the first systematic review and meta-analysis to examine the cardiorespiratory health effects of gaseous AAP in LMICs exclusively. It also included enough studies to perform subgroup analysis demonstrating clear spatial variations in results as well as variations by outcome and co-pollutant adjustment. However, several limitations should be noted, for numerous pooled estimates heterogeneity was high the sources of which could not always be determined this may be due to the aggregation of cardiorespiratory outcomes of which we were unable to fully stratify due to low study numbers. Although we only included studies with clinically confirmed health outcomes an inherent limitation regarding health outcome data quality in LMICs should also be noted. Furthermore, the use of fixed site monitoring as a surrogate for individual exposure is a common yet considerable limitation seen in all included studies. Further research is greatly required with an emphasis on more accurate exposure classification through assignment of individual exposure. As mortality displacement cannot be ruled out in explaining at least some of the observed associations it would be beneficial to also include moving average lags over greater time periods, however these were not available in the included studies. A further limitation is that although adjustment for additional pollutants was performed attenuating several the observed associations, due to the heterogeneous nature of AAP the degree to which AAP components and their subsequent health effects can be “isolated”, makes it particularly difficult to determine the effects of individual pollutants on health with further research is required. Finally, we did not include panel studies (due to their typical focus on smaller sub groups of populations) and only studies published in the English language were included.

## Conclusion

This study has demonstrated how gaseous AAP is associated with a range of cardiorespiratory outcomes within LMICs, with clear spatial variations apparent. Although some obtained results from LMIC regions are similar in magnitude to those from within HICs, they are far from consistent enough to warrant extrapolation of results from HICs into LMIC regions. Clear spatial variations have been observed down to a city level within LMICs demonstrating the spatial heterogeneity in gaseous AAP and the associated health effects. Therefore, such extrapolation of results is unfeasible with potential to misalign AAP policy making. Further research is required within LMICs exclusively (particularly those not encompassed by this review such as Sub-Saharan Africa and South Asia) to fully examine the health effects of gaseous AAP. Only then can the growing burden of gaseous AAP in LMICs be adequately addressed and reduced.

### Additional file

Additional file 1: Supplementary materials.ᅟ(PDF 1875 kb)

## Appendix 1

### Search strategy

**Table 1:** The cardiorespiratory health effects of acute exposure to gaseous ambient air pollution within low and/or middle income countries (LMICs)

Places to search for information	PubMed
	EMBASE
	Web of Science
	Proquest
	Global Health
	LILACs

### Search string

(((((“Air pollution*” “nitrogen dioxide” OR “nitric oxide” OR “sulfur dioxide” OR “ozone” OR “carbon monoxide”) AND ((“time series” OR “timeseries” OR “time-series” OR “case crossover” OR “case control” OR “cohort” OR “cross sectional”))) AND ((“mortality” OR “death” OR “admission” OR “hospital admission” OR “emergency room” OR “accident & emergency”))) AND (“developing country” OR “Afghanistan” OR “Guinea” OR “Rwanda” OR “Benin” OR “Guinea-Bissau” OR “Senegal” OR “Benin” OR “Guinea-Bissau” OR “Senegal” OR “Burkina Faso” OR “Haiti” OR “Sierra Leone” OR “Burundi” OR “Korea Dem. People’s Rep*” OR “Somalia” “Central African Republic” OR “Liberia” OR “South Sudan” OR “Chad” OR “Madagascar” OR “Tanzania” OR “Comoros” OR “Malawi” OR “Togo” OR “Congo Dem. Rep” OR “Mali” OR “Uganda” OR “Eritrea” OR “Mozambique” OR “Zimbabwe” OR “Ethiopia” OR “Nepal” OR “Gambia” OR “Niger” OR “Armenia” OR “Kiribati” OR “Soloman Islands” OR “Bangladesh” OR “Kosovo” OR “Sri Lanka” OR “Bhutan” OR “Kyrgyz*” OR “Sudan” OR “Bolivia” OR “Lao” OR “Swaziland” OR “Cabo Verde” OR “Lesotho” OR “Syria*” OR “Cambodia” OR “Mauritania” OR “Tajikistan” OR “Cameroon” OR “Micronesia” OR “Timor-Leste” OR “Congo. Rep” OR “Moldova” OR “Tonga” OR “Cote d’Ivoire” OR “Mongolia” OR “Tunisia” OR “Dijibouti” OR “Morocco” OR “Ukraine” OR “Egypt, Arab Rep” OR “Myanmar” OR “Uzbekistan” OR “El Salvador” OR “Nicaragua” OR “Vanuatu” OR “Ghana” OR “Nigeria” OR “Vietnam” OR “Guatemala” OR “Pakistan” OR “West Bank and Gaza” OR “Honduras” OR “Papua New Guinea” OR “Yemen*” OR “India” OR “Philippines” OR “Zambia” OR “Indonesia OR” “Samoa” OR “Kenya” OR “Sao Tome and Principe” OR “Albania” OR “Ecuador” OR “Montenegro” OR “Algeria” OR “Fiji” OR “Namibia” OR “American Samoa” OR “Gabon” OR “Palau” OR “Angola” OR “Georgia” OR “Panama” OR “Argentina” OR “Grenada” OR “Paraguay” OR “Azerbaijan” OR “Guyana” OR “Peru” OR “Belarus” OR “Iran, Islamic Rep” OR “Romania” OR “Belize” OR “Iraq” OR “Russia*” OR “Bosnia and Herzegovina” OR “Jamaica” OR “Serbia” OR “Botswana” OR “Jordan” OR “South Africa” OR “Brazil” OR “Kazakhstan” OR “St. Lucia” OR “Bulgaria” OR “Lebanon” OR “St Vincent and the Grenadines” OR “China” OR “Libya” OR “Suriname” OR “Colombia” OR “Macedonia” OR “Thailand” OR “Costa Rica” OR “Malaysia” OR “Turkey” OR “Cuba” OR “Maldives” OR “Turkmenistan” OR “Dominica” OR “Marshall Islands” OR “Tuvalu” OR “Dominican Republic” OR “Mauritius” OR “Venezuela” OR “Equatorial Guinea” OR “Mexico” OR “Africa” OR “Caribbean Region” OR “Central America” OR “Latin America” OR “South America” OR “Asia”))

## Appendix 2

### Risk of bias

**Table 2:** Exposure assessment bias

	Exposure Metric	Risk of exposure assessment bias
A	Exposure as a surrogate for air pollution (e.g. distance to road, personal recall)	High
A	Use of single fixed monitoring system	High
A	Use of multiple fixed monitoring systems	≥3 Moderate
		< 3 High
B	Personal exposure	Low
B	Atmospheric dispersion models	Moderate
B	Land use regression models	Moderate
B	Satellite/remote sensing	Moderate

Combination of > 2 of the above (providing at least one B measure) = low risk of bias

## Abbreviations

AAP: Ambient air pollution
CO: Carbon monoxide
HIC: High income country
LMIC: Low and middle income country
NO_x_: Nitrogen oxides
O_3_: Ozone
PM_10_: Particulate matter (< 10 μm)
PM_2.5_: Particulate matter (< 2.5 μm)
ppm: Parts per million
SO_2_: Sulfur dioxide
