# Supplementary material for: Cardiorespiratory health effects of gaseous ambient air pollution exposure in low and middle income countries: a systematic review and meta-analysis
Source: Environ Health. 2018 Apr 18;17:41. doi: 10.1186/s12940-018-0380-3 (PMC5907176; doi:10.1186/s12940-018-0380-3)
Supplement: Supplementary file 1 — Supplementary materials.ᅟ(PDF 1875 kb) [file 12940_2018_380_MOESM1_ESM.pdf]

Supplementary Data

Part A – Converting effect estimates

Converting estimates from IQR to10 µg/m^3.

Example;

Each IQR increase = 6.31% excess risk

IQR = 29

To get % excess risk per 10 µg/m^3;

29/100 = 0.29 10/0.29=34.48 (this is the conversion factor)

(6.31/100)*34.48 = 2.18 % excess risk per 10 µg/m^3;

Converting estimates from beta coefficients per IQR to % excess risk (ER) per 10 µg/m^3.

IQR = 15.90µg/m^3.

B=0.004 SE (0.002)

HR = exp(0.004) = 1.004 per 15.90µg/m^3

0.4% ER per 15.90µg/m3

15.9/100 = 0.159 10/0.159 = 62.89 (this is the conversion factor)

(0.4/100)*62.89 = 0.25156

Part B – Included study characteristics

Table 1: Cohort studies

Author	Year	Study Period	City	Region	Outcome	$PM_{2.5}$	PM_{10}	SO_2	NOx	O_3	TSP
**Zhang, P.**	2011	1990-2009	Shenyang	East Asia and China	M (CV)	No	Yes	Yes	Yes	No	No
**Cao, J.**	2011	1991-2000	Multi-city	East Asia and China	M (CV & R)	No	No	Yes	Yes	No	Yes
**Dong, G.**	2011	1998-2009	Shenyang	East Asia and China	M (R)	No	Yes	Yes	Yes	No	No

Abbreviations: M = Mortality, A = Admissions; CV=Cardiovascular; R=Respiratory;

Table 2: Case-crossover studies

Author	Year	Study Period	City	Region	Outcome	$PM_{2.5}$	PM_{10}	SO_2	NOx	O_3	TSP	CO
**Qian, Z.**	2013	2003-2008	Shanghai	East Asia and Pacific	M(CV)	No	Yes	Yes	Yes	No	No	No
**Guo, Y.**	2009	2004-2006	Beijing	East Asia and Pacific	A (CV)	Yes	No	Yes	Yes	No	No	No
**Xiang, H.**	2013	2006-2008	Wuhan	East Asia and Pacific	A (CV)	No	Yes	Yes	Yes	No	No	No
**Liu, Y.**	2015	2006-2009	Anshan	East Asia and Pacific	M(CV)	Yes	No	Yes	Yes	No	No	No
**Huang, F.**	2016	2013-2014	Beijing	East Asia and Pacific	A (CV)	Yes	Yes	No	Yes	No	No	Yes

Abbreviations: M = Mortality, A = Admissions; CV=Cardiovascular; R=Respiratory;

Table 3: Time-series studies

Author	Year	Study Period	City	Region	Outcome	$PM_{2.5}$	PM_{10}	SO_2	NOx	O_3	TSP	CO
**Abrutsky, R.**	2012	1996-2008	Buenos Aires	Latin America and Caribbean	M (CV)	No	No	No	No	No	No	Yes
**Capraz, O.**	2016	2007-2012	Istanbul	Europe and Central Asia	M (CV&R)	No	Yes	Yes	Yes	No	Yes	No
**Chen, G**	2008	2001-2004	Shanghai	East Asia and Pacific	M (CV&R)	No	Yes	Yes	Yes	No	No	No
**Chen, R.**	2010	2005-2007	Shanghai	East Asia and Pacific	A (CV&R)	No	Yes	Yes	No	Yes	Yes	No
**Chen, R.**	2013	1996-2008	Multi-City	East Asia and Pacific	M (CV&R)	No	Yes	Yes	Yes	No	No	No
**Costa, A.F.**	2016	2001-2011	Sao Paulo	Latin America and Caribbean	M (CV&R)	No	Yes	No	Yes	No	No	Yes
**de Freitas**	2010	2001-2006	Vitoria	Latin America and Caribbean	A (CV)	No	Yes	No	Yes	Yes	No	No
**Gharehchachi, E.**	2013	2008-2010	Shiraz	Middle East and North Africa	A (CV&R)	No	Yes	Yes	Yes	No	No	No
**Hosseinpoor, A.R**	2005	1996-2001	Tehran	Middle East and North Africa	A (CV)	No	Yes	Yes	Yes	Yes	No	Yes
**Kan, H.**	2003	2000-2001	Shanghai	East Asia and Pacific	M (CV&R)	No	Yes	Yes	Yes	No	No	No
**Kan, H.**	2010	2001-2004	Shanghai	East Asia and Pacific	M (CV&R)	No	Yes	Yes	Yes	Yes	No	No
**Kan, H.**	2003	2001-2002	Shanghai	East Asia and Pacific	M (CV)	No	Yes	Yes	Yes	No	No	No
**Kan, H.**	2008	2001-2004	Shanghai	East Asia and Pacific	M (CV&R)	No	Yes	Yes	Yes	Yes	No	No
**Kan, H.**	2010	1999-2004	Multi-City	East Asia and Pacific	M (CV&R)	No	Yes	Yes	Yes	Yes	No	No
**Meng, X.**	2013	1996-2008*	Multi-City	East Asia and Pacific	M (R)	No	Yes	Yes	Yes	No	No	No
**Nascimento, L.F.**	2012	2007-2008	San Jose dos	Latin America	A (C)	No	Yes	Yes	No	Yes	No	No

			Campos	and Caribbean								
**Phung, D.**	2016	2004-2007	Ho Chi Minh City	East Asia and Pacific	A (CV&M)	No	Yes	Yes	Yes	Yes	No	No
**Qian, Z.**	2010	2000-2004	Wuhan	East Asia and Pacific	M (CV&R)	No	Yes	Yes	Yes	Yes	No	No
**Qian, Z.**	2007	2000-2004	Wuhan	East Asia and Pacific	M (CV&R)	No	No	Yes	Yes	Yes	No	No
**Romieu, I.**	2012	1997-2005	Multi-City	Latin America and Caribbean	M (CV&R)	No	Yes	No	No	Yes	No	No
**Stojic, S.S**	2016	2009-2014	Belgrade	Europe and Central Asia	M (CV&R)	No	Yes	Yes	Yes	No	No	No
**Tao, Y.**	2012	2006-2008	Multi-City	East Asia and Pacific	M (CV&R)	No	Yes	No	Yes	Yes	No	No
**Tong, L.**	2014	2008-2011	Tianjin	East Asia and Pacific	A (CV)	No	Yes	Yes	No	No	No	No
**Wong, C.M**	2008	1999-2004	Multi-City	East Asia and Pacific	M (CV&R)	No	Yes	Yes	Yes	No	No	No
**Xie, J.**	2014	2010-2012	Shanghai	East Asia and Pacific	A (CV)	No	Yes	Yes	Yes	No	No	No
**Yu, I.T.S**	2012	2006-2009	Guangzhou	East Asia and Pacific	M (CV&R)	No	Yes	Yes	Yes	No	No	No
**Zhang, F.**	2011	2003-2008	Beijing	East Asia and Pacific	M (CV&R)	No	Yes	Yes	Yes	No	No	No
**Zhang, Y**	2015	2009-2011	Beijing	East Asia and Pacific	A (CV&R)	No	Yes	Yes	Yes	No	No	No
**Zhang, Y**	2006	2001-2004	Shanghai	East Asia and Pacific	M (CV&R)	No	No	No	No	Yes	No	No
**Zhang, Z.L**	2014	2008-2011	Guangzhou	East Asia and Pacific	A (CV&R)	No	Yes	Yes	Yes	No	No	No
**Amancio, C.T**	2014	2005-2009	San Jose dos Campos	Latin America and Caribbean	M (CV&R)	No	Yes	Yes	No	No	No	No
**Arbex, M.A**	2009	2001-2003	Sao Paulo	Latin America and Caribbean	A (R)	No	Yes	Yes	No	No	No	Yes
**Tao, Y.**	2011	2006-2008	Multi-City	East Asia and Pacific	M (CV&R)	No	Yes	Yes	Yes	No	No	Yes
**Tao, Y.**	2014	2001-2005	Lanzhou	East Asia and Pacific	A (R)	No	Yes	Yes	Yes	No	No	No
**Cai, J.**	2014	2005-2011	Shanghai	East Asia and	A (R)	No	Yes	Yes	Yes	No	No	No

				Pacific								
**Leitte, A.M**	2011	2004-2006	Beijing	East Asia and Pacific	A (R)	No	Yes	Yes	Yes	No	No	No
**Li, G.**	2016	2002-2006	Tianjin	East Asia and Pacific	M (CV)	No	Yes	Yes	Yes	No	No	No
**Mahiyuddin, W.R.V**	2013	2000-2006	Klang-Valley	East Asia and Pacific	M (CV&R)	No	Yes	No	Yes	Yes	No	No
**Su, C.**	2016	2007-2008	Beijing	East Asia and Pacific	A (CV &R)	Yes	Yes	Yes	Yes	No	No	No
**Yang, Y.**	2015	2009-2010	Beijing	East Asia and Pacific	M (R)	No	Yes	No	Yes	No	No	No
**Zheng, S.**	2013	2001-2005	Lanzhou	East Asia and Pacific	A (C)	No	Yes	Yes	Yes	No	No	No
**Yang, C.**	2012	2006-2008	Suzhou	East Asia and Pacific	M (CV&R)	No	No	No	No	Yes	No	No
**Lin, C.**	2003	1994-1995	Sao Paulo	Latin America and Caribbean	A (CV)	No	Yes	Yes	No	No	No	Yes
**Zahari, M.**	2014	2004-2009	Multi-City		A (R)	No	Yes	No	No	No	No	Yes
**Yang, Y.**	2013	2009-2010	Beijing	East Asia and Pacific	M (CV&R)	No	Yes	No	Yes	No	No	No
**Sharovsky, R.**	2004	1996-1998	Sao Paulo	Latin America and Caribbean	M (R)	No	Yes	Yes	No	No	No	Yes
**Chen, R.**	2012	2001-2008	Multi-City	East Asia and Pacific	M (C)	No	Yes	Yes	Yes	No	No	No
**Chen, R.**	2012	2001-2008	Multi-City	East Asia and Pacific	M (CV&R)	No	No	No	Yes	No	No	No
**Chen, R.**	2012	2001-2010	Multi-City	East Asia and Pacific	M (CV&R)	No	No	Yes	No	No	No	No
**Abrutsky, R.**	2013	2005-2009	Multi-City	Latin America and Caribbean	M (R)	No	No	No	No	No	No	Yes
**Guo, L.J**	2014	2010-2011	Shanghai	East Asia and Pacific	A (R)	No	Yes	Yes	Yes	No	No	No
**Zhao, A.**	2014	2010-2011	Shanghai	East Asia and Pacific	A (C)	No	Yes	Yes	Yes	No	No	No
**Gavinier, S.**	2014	2007-2011	Sao Paulo	Latin America and Caribbean	A (CV)	No	Yes	No	Yes	Yes	No	No

Abbreviations: M = Mortality, A = Admissions; CV=Cardiovascular; R=Respiratory;

Table 4: Both case-crossover and time-series studies

Author	Year	Study Period	City	Region	Outcome	PM2.5	PM10	SO2	NOx	O3	TSP	CO
**Guo, Y.**	2010	2005-2007	Tianjin	East Asia and Pacific	M (CV)	No	Yes	Yes	Yes	No	No	No
**Winchman, J.**	2012	2001-2006	Cape Town	Africa	M (CV & R)	No	Yes	Yes	Yes	No	No	No

Abbreviations: M = Mortality, A = Admissions; CV=Cardiovascular; R=Respiratory;

Table 5: Results from identified cohort studies

Author	Year	Study Period	Pollutant	Outcome	Specific Outcome	RR (95% CI)	Adjusted for	Single or multi-pollutant model
Zhang, P.	2011	1998-2009	SO_2	Cardiovascular Mortality	-	0.96 (0.92 - 1.01)	Adjusted[1]	Single
				Cardiovascular Mortality	I60-I69	0.95 (0.90 - 1.00)	Adjusted[1]	Single
			NO_2	Cardiovascular Mortality	-	2.46 (2.31 – 2.63)	Adjusted [1]	Single
				Cardiovascular Mortality	I60-I69	2.44 (2.27 – 2.62)	Adjusted[1]	Single
Cao, J.	2011	1991-2000	SO_2	Respiratory Mortality		1.015 (1.003, 1.028)	Adjusted[2]	Single
				Respiratory Mortality	Lung Cancer	1.040 (1.024, 1.056)	Adjusted[2]	Single
				Cardiovascular Mortality		1.048 (1.040, 1.056)	Adjusted[2]	Single
				Respiratory Mortality		1.032 (1.018, 1.047)	Adjusted[3]	Single
				Respiratory Mortality	Lung Cancer	1.042 (1.023, 1.062)	Adjusted[3]	Single
				Cardiovascular Mortality		1.032 (1.023, 1.040)	Adjusted[3]	Single
				Respiratory Mortality		1.032 (1.017, 1.047)	Adjusted[3]	Multi (TSP)
				Respiratory Mortality	Lung Cancer	1.041 (1.021, 1.061)	Adjusted[3]	Multi (TSP)
				Cardiovascular Mortality		1.031 (1.022, 1.040)	Adjusted[3]	Multi (TSP)
				Respiratory Mortality		1.031 (1.016, 1.046)	Adjusted[3]	Multi (NO_X)
				Respiratory Mortality	Lung Cancer	1.041 (1.021, 1.061)	Adjusted[3]	Multi (NO_X)
				Cardiovascular Mortality		1.031 (1.022, 1.040)	Adjusted[3]	Multi (NO_X)
			NO_X	Respiratory Mortality		1.017 (0.987, 1.048)	Adjusted[2]	Single
				Respiratory Mortality	Lung Cancer	1.016 (0.980, 1.053)	Adjusted[2]	Single
				Cardiovascular Mortality		1.027 (1.010, 1.043)	Adjusted[2]	Single
				Respiratory Mortality		1.026 (0.998, 1.056)	Adjusted[3]	Single
				Respiratory Mortality	Lung Cancer	1.027 (0.991, 1.065)	Adjusted[3]	Single
				Cardiovascular Mortality		1.023 (1.006, 1.041)	Adjusted[3]	Single
				Respiratory Mortality		1.021 (0.990, 1.53)	Adjusted[3]	Multi (TSP)
				Respiratory Mortality	Lung Cancer	1.017 (0.979, 1.057)	Adjusted[3]	Multi (TSP)
				Cardiovascular Mortality		1.015 (0.996, 1.033)	Adjusted[3]	Multi (TSP)
				Respiratory Mortality		1.015 (0.985, 1.046)	Adjusted[3]	Multi (SO_2)
				Respiratory Mortality	Lung Cancer	1.013 (0.974, 1.053)	Adjusted[3]	Multi (SO_2)
				Cardiovascular Mortality		1.012 (0.994, 1.030)	Adjusted[3]	Multi (SO_2)
Dong, G.	2011	1998-2009	SO_2	Respiratory Mortality	-	1.04 (0.97 – 1.12)	Adjusted[4]	Single
			NO_2	Respiratory Mortality	-	2.97 (2.69 – 3.27)	Adjusted[4]	Single

[1] Adjusted model: adjusted for age, sex, BMI, educational level, smoking habits, personal income, occupational exposure, and exercise frequency.
[2] Adjusted model: adjusted for age and sex
[3] Adjusted model: adjusted for age, sex, BMI, physical activity, education, smoking status, age at starting to smoke, years smoked, cigarettes per day, alcohol intake, and hypertension.
[4] Adjusted model: adjusted for age, gender, educational level, smoking status, personal income, occupational exposure, BMI and exercise

Nitrogen Dioxide Funnel Plots

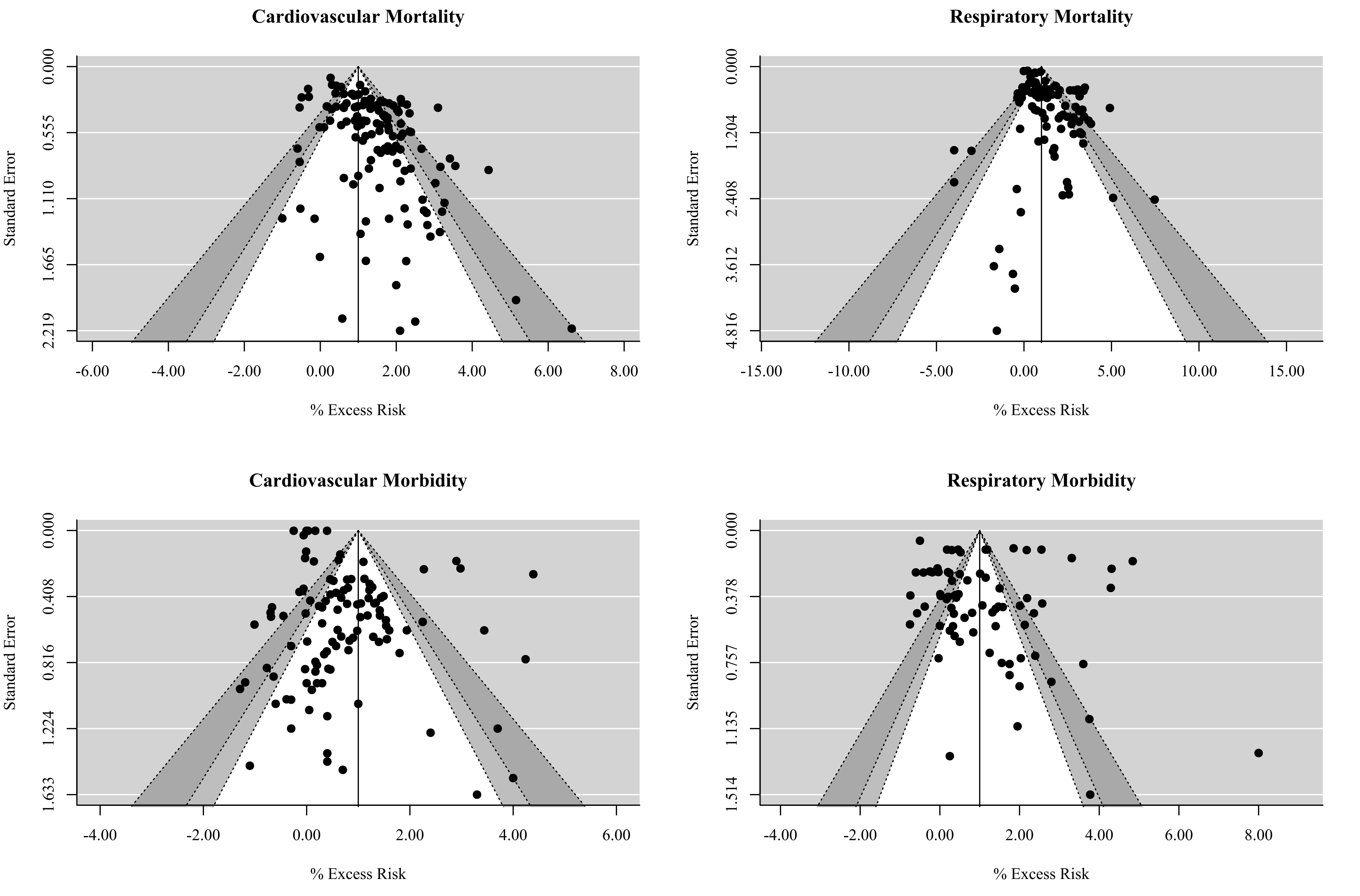

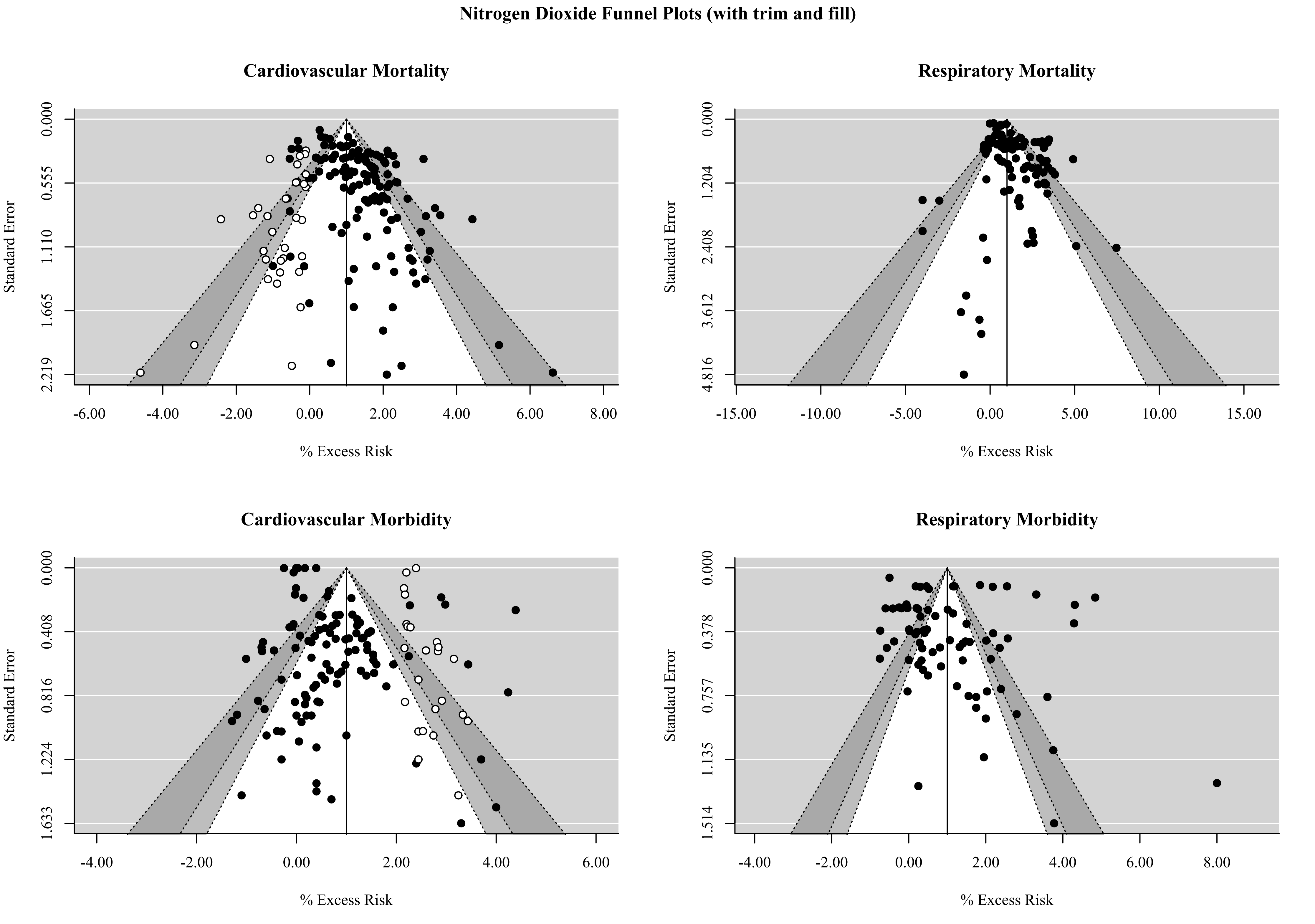
Nitrogen Dioxide Funnel Plots (with trim and fill)
Cardiovascular Mortality
Standard Error
0.000
0.555
1.110
1.665
2.219
-6.00
-4.00
-2.00
0.00
2.00
4.00
6.00
8.00
% Excess Risk
Respiratory Mortality
Standard Error
0.000
1.204
2.408
3.612
4.816
-15.00
-10.00
-5.00
0.00
5.00
10.00
15.00
% Excess Risk
Cardiovascular Morbidity
Standard Error
0.000
0.408
0.816
1.224
1.633
-4.00
-2.00
0.00
2.00
4.00
6.00
% Excess Risk
Respiratory Morbidity
Standard Error
0.000
0.378
0.757
1.135
1.514
-4.00
-2.00
0.00
2.00
4.00
6.00
8.00
% Excess Risk

Sulphur Dioxide Funnel Plots

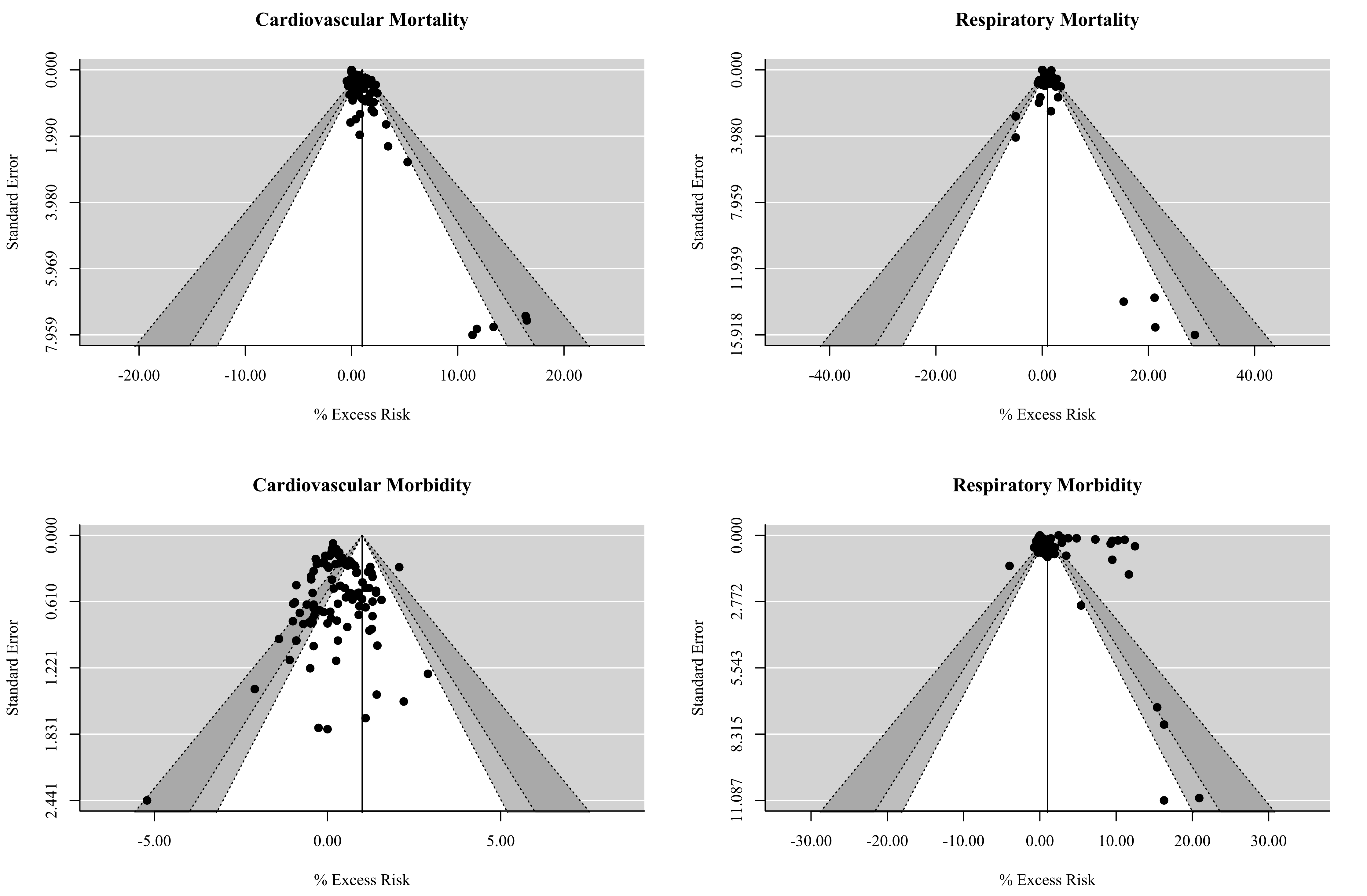

Sulphur Dioxide Funnel Plots (with trim and fill)

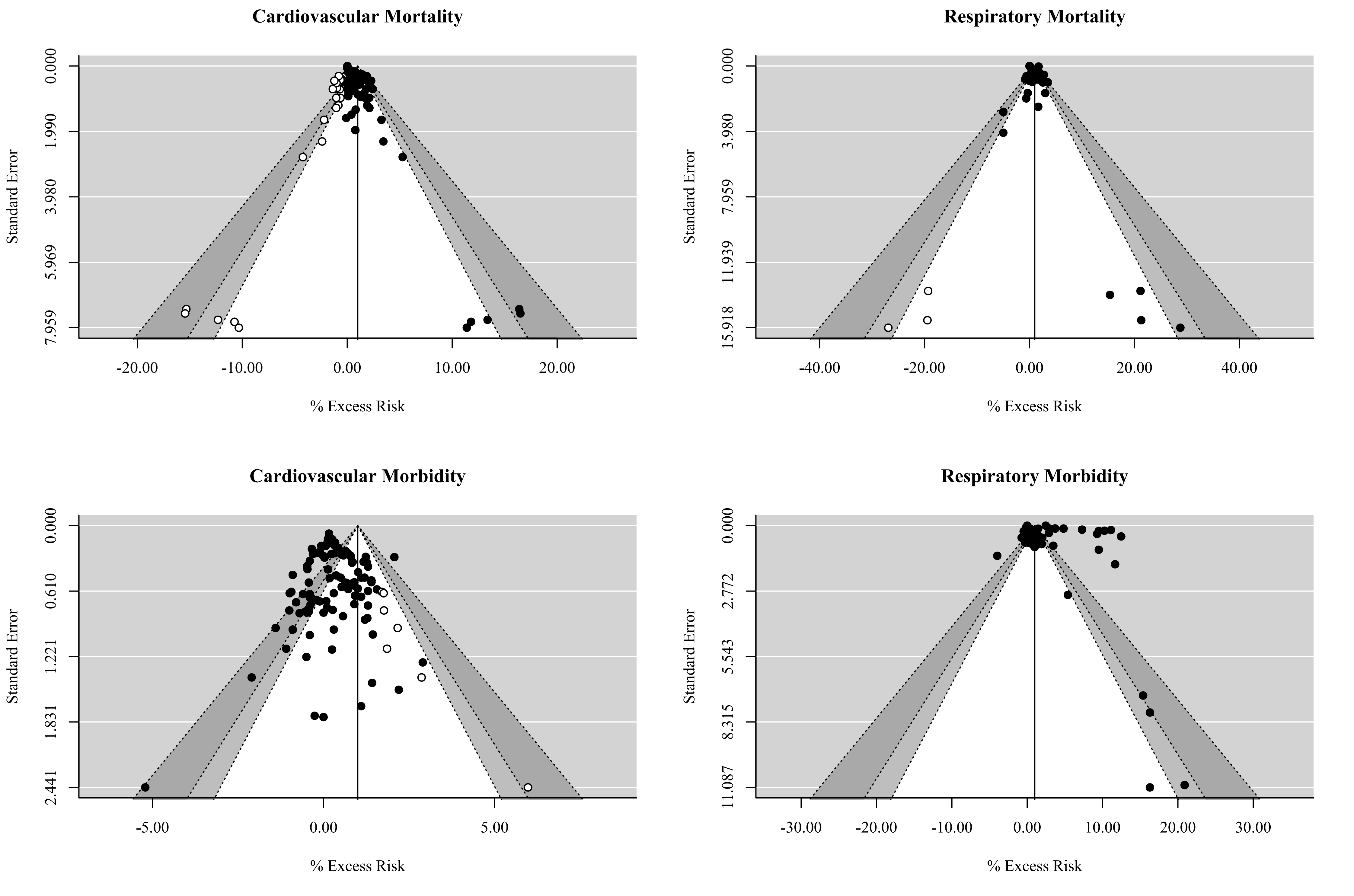

Carbon Monoxide Funnel Plots

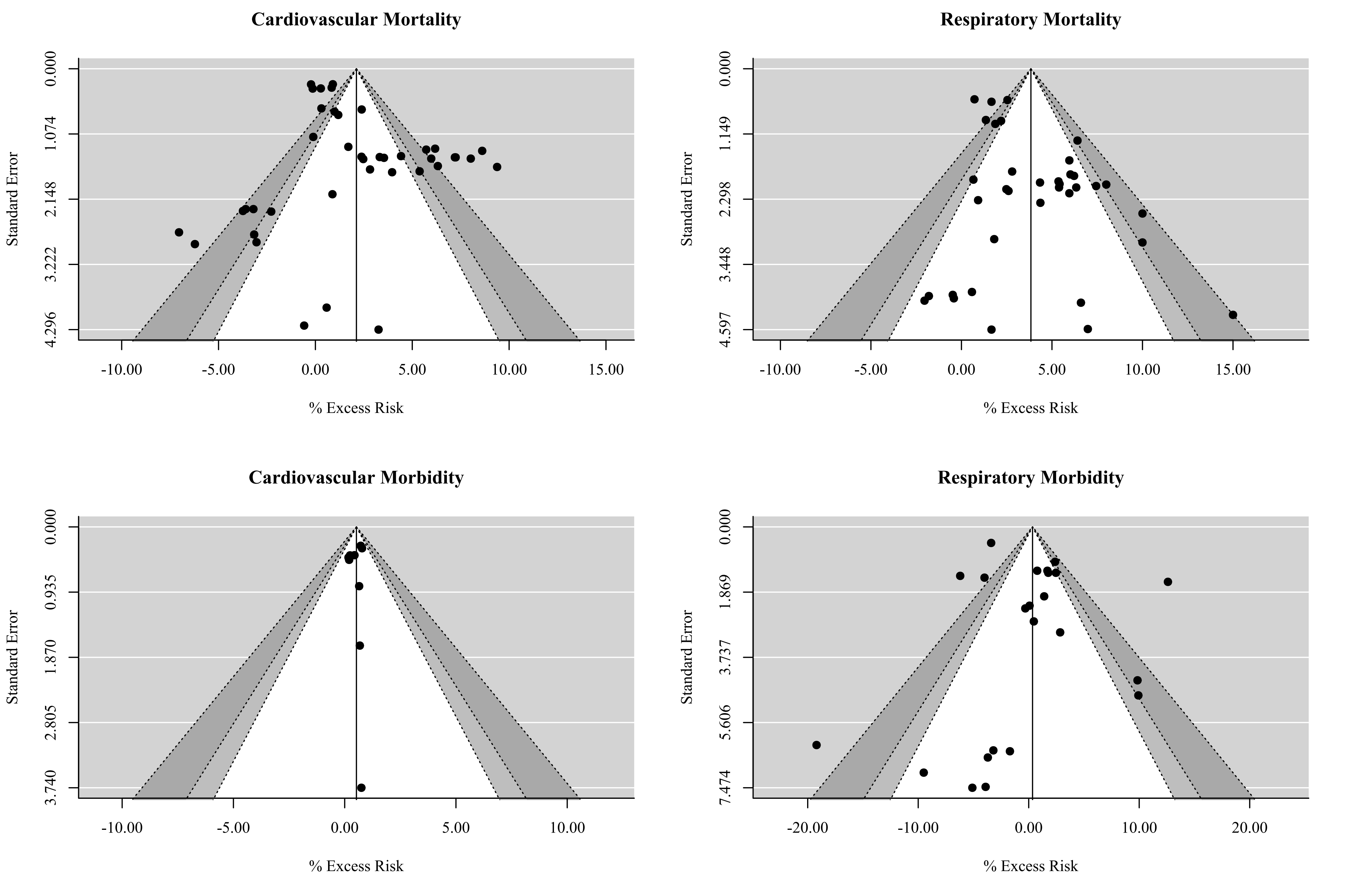

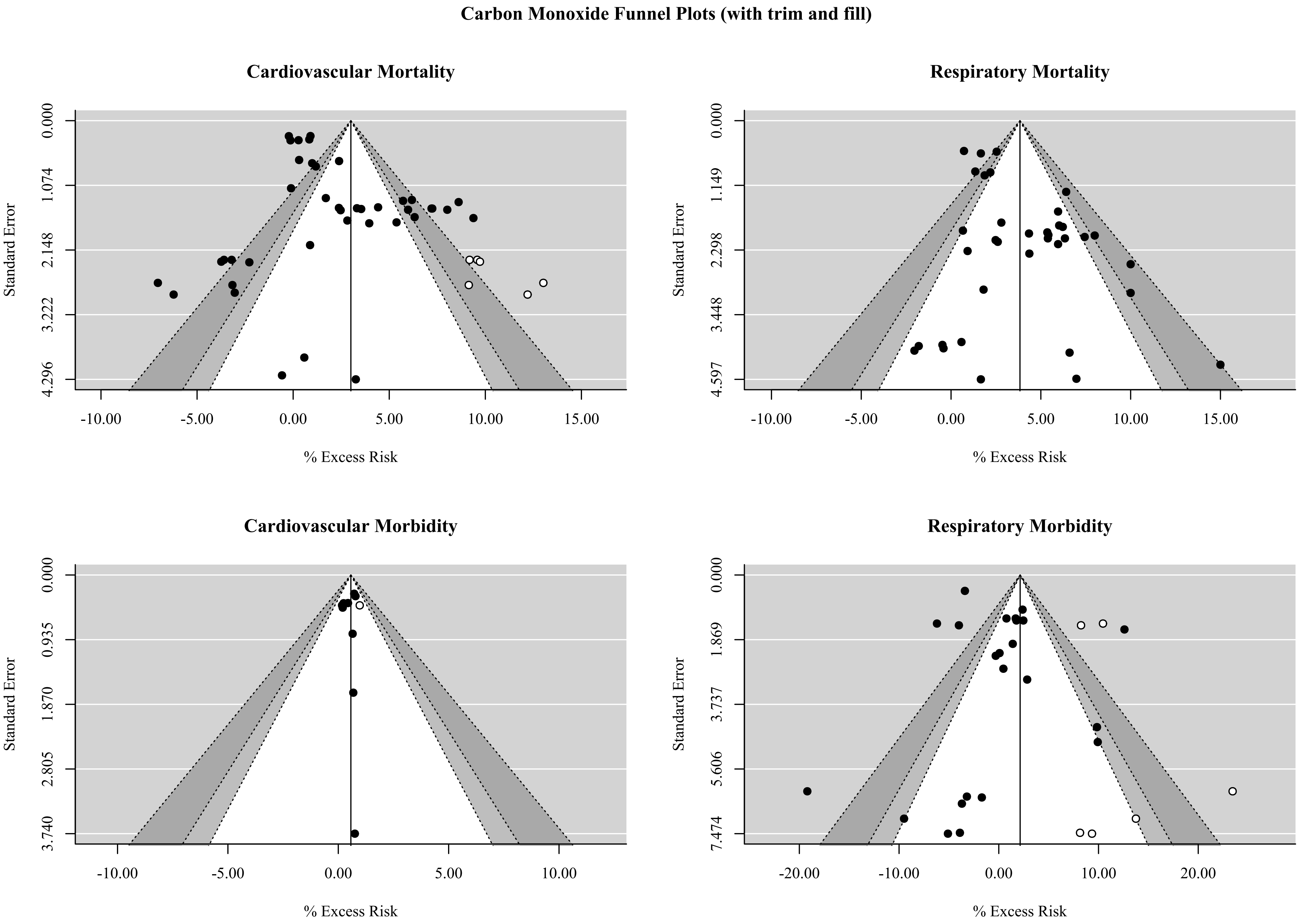

Carbon Monoxide Funnel Plots (with trim and fill)
Cardiovascular Mortality
Respiratory Mortality
Cardiovascular Morbidity
Respiratory Morbidity
Standard Error
% Excess Risk
0.000
1.074
2.148
3.222
4.296
1.149
2.298
3.448
4.597
0.935
1.870
2.805
3.740
1.869
3.737
5.606
7.474
-10.00
-5.00
0.00
5.00
10.00
15.00
-20.00
20.00

Ozone Funnel Plots

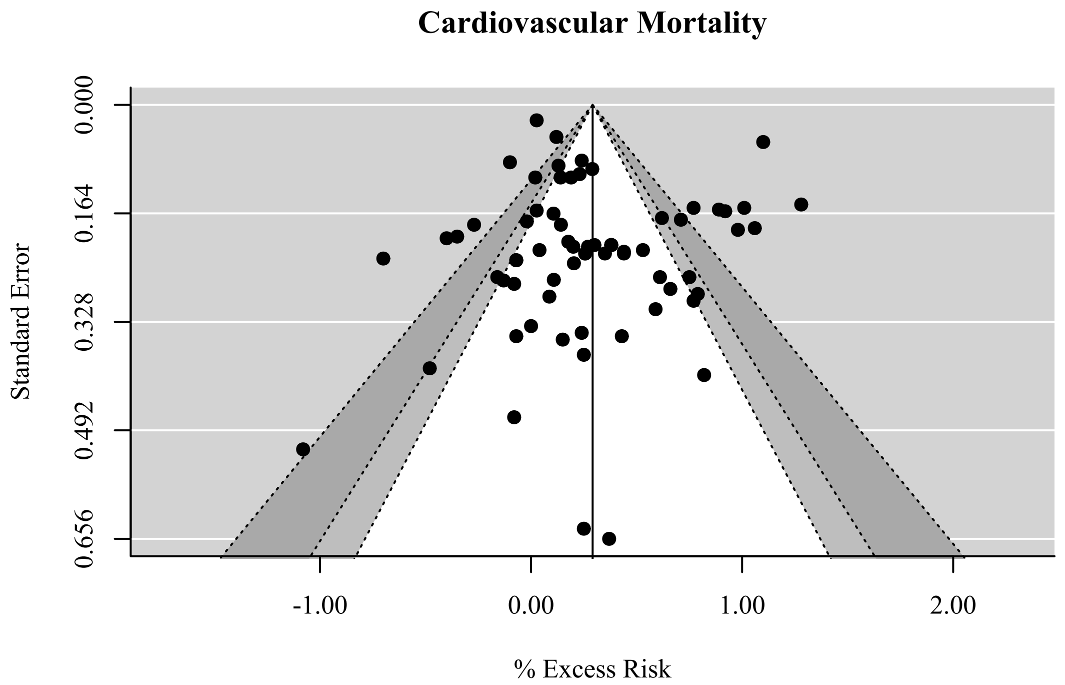

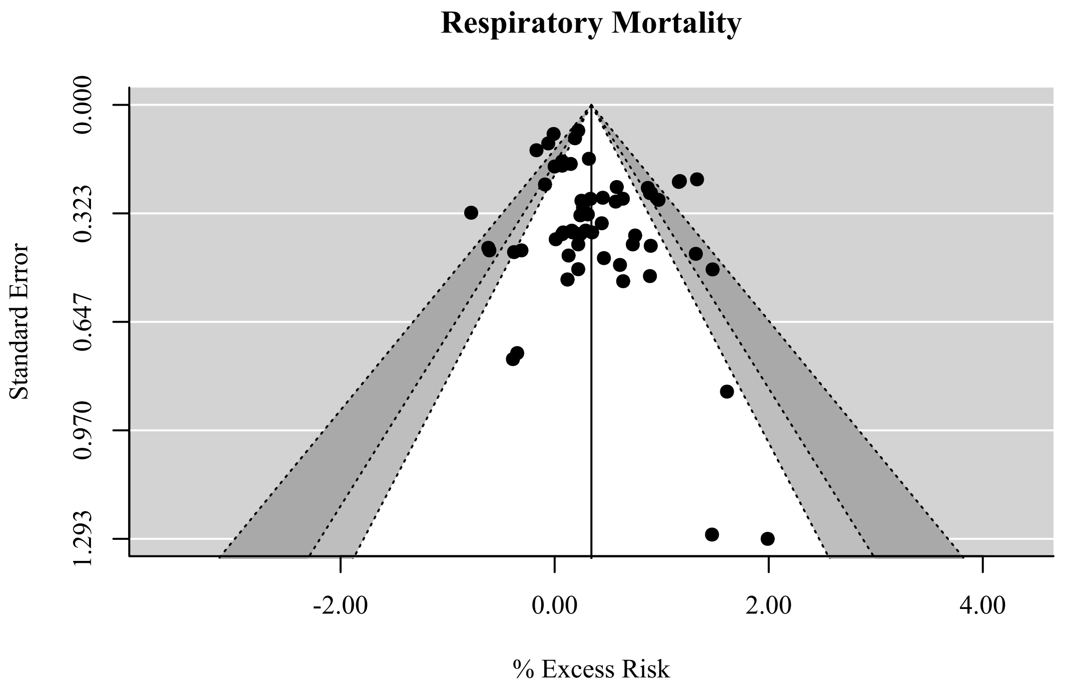

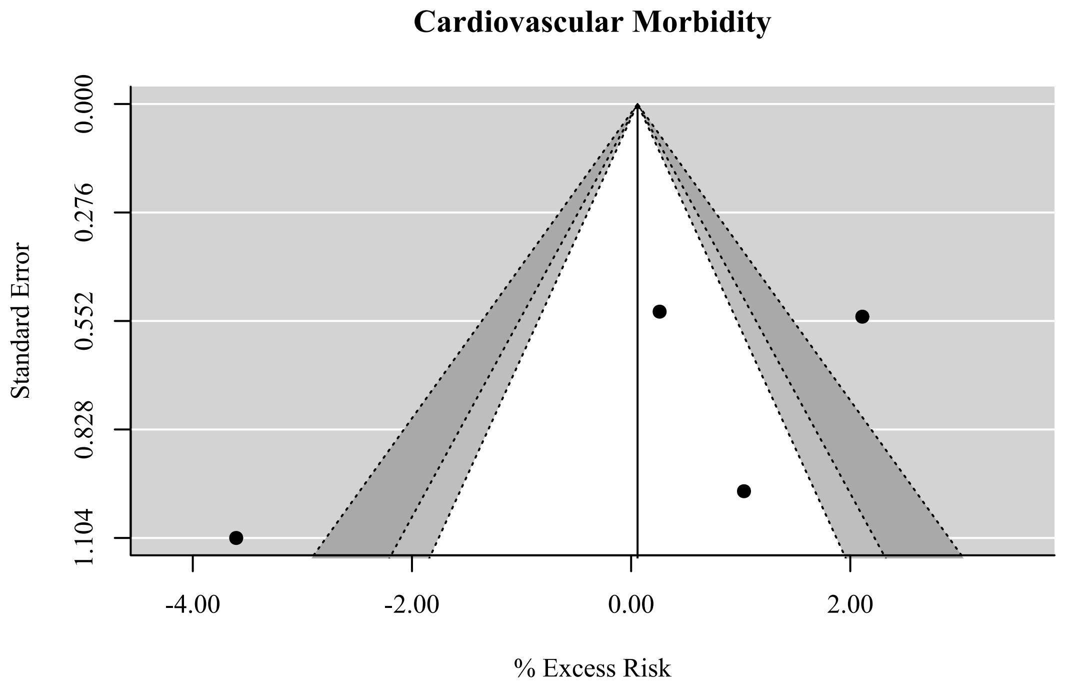

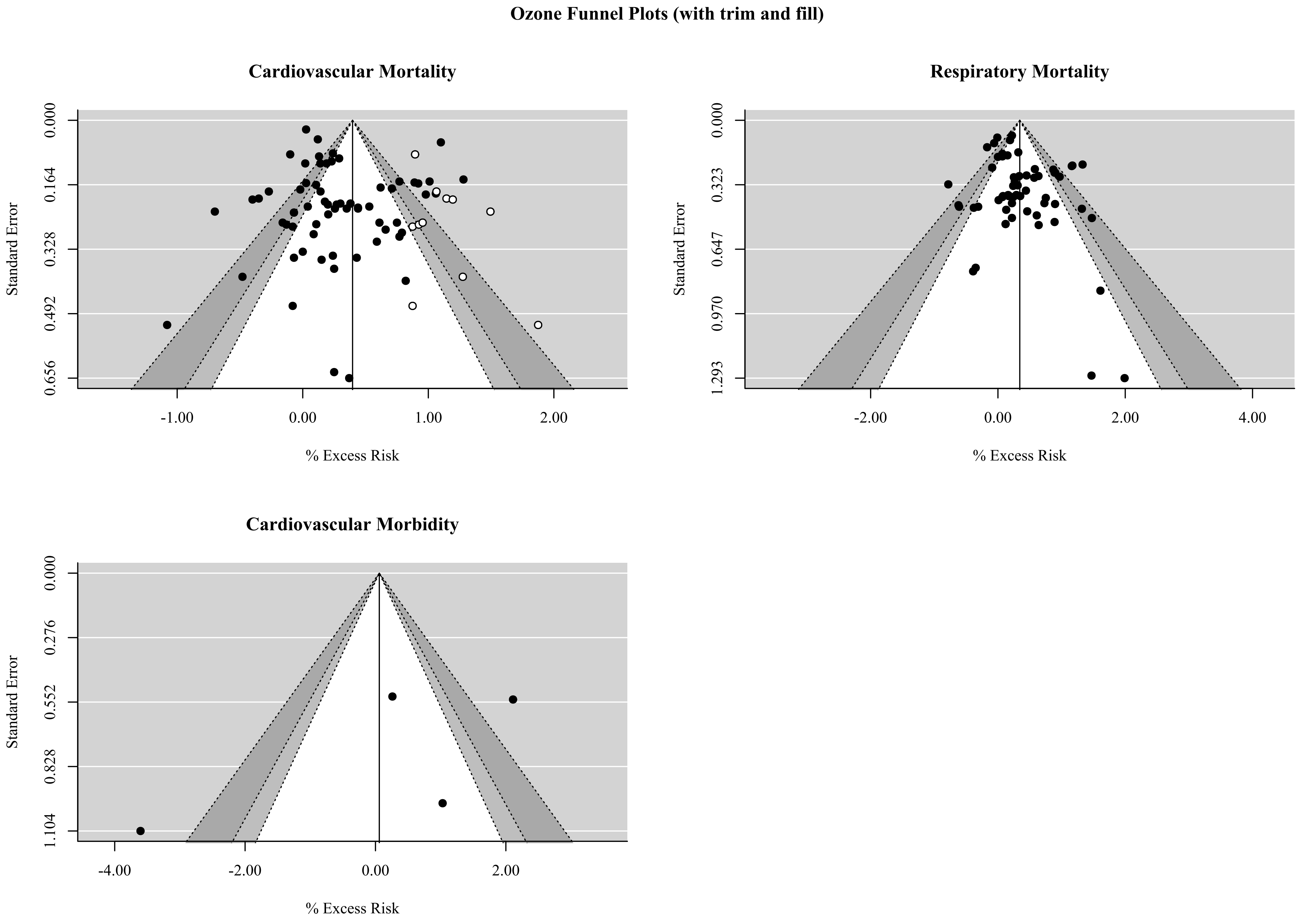
Ozone Funnel Plots (with trim and fill)
Cardiovascular Mortality
Standard Error
0.000
0.164
0.328
0.492
0.656
-1.00
0.00
1.00
2.00
% Excess Risk
Respiratory Mortality
Standard Error
0.000
0.323
0.647
0.970
1.293
-2.00
0.00
2.00
4.00
% Excess Risk
Cardiovascular Morbidity
Standard Error
0.000
0.276
0.552
0.828
1.104
-4.00
-2.00
0.00
2.00
% Excess Risk

PRISMA 2009 Checklist

Section/topic	#	Checklist item	Reported on page #
**TITLE**			
Title	1	Identify the report as a systematic review, meta-analysis, or both.	1
**ABSTRACT**			
Structured summary	2	Provide a structured summary including, as applicable: background; objectives; data sources; study eligibility criteria, participants, and interventions; study appraisal and synthesis methods; results; limitations; conclusions and implications of key findings; systematic review registration number.	3
**INTRODUCTION**			
Rationale	3	Describe the rationale for the review in the context of what is already known.	4
Objectives	4	Provide an explicit statement of questions being addressed with reference to participants, interventions, comparisons, outcomes, and study design (PICOS).	4
**METHODS**			
Protocol and registration	5	Indicate if a review protocol exists, if and where it can be accessed (e.g., Web address), and, if available, provide registration information including registration number.	4
Eligibility criteria	6	Specify study characteristics (e.g., PICOS, length of follow-up) and report characteristics (e.g., years considered, language, publication status) used as criteria for eligibility, giving rationale.	5
Information sources	7	Describe all information sources (e.g., databases with dates of coverage, contact with study authors to identify additional studies) in the search and date last searched.	5
Search	8	Present full electronic search strategy for at least one database, including any limits used, such that it could be repeated.	5
Study selection	9	State the process for selecting studies (i.e., screening, eligibility, included in systematic review, and, if applicable, included in the meta-analysis).	5
Data collection process	10	Describe method of data extraction from reports (e.g., piloted forms, independently, in duplicate) and any processes for obtaining and confirming data from investigators.	5
Data items	11	List and define all variables for which data were sought (e.g., PICOS, funding sources) and any assumptions and simplifications made.	5
Risk of bias in individual studies	12	Describe methods used for assessing risk of bias of individual studies (including specification of whether this was done at the study or outcome level), and how this information is to be used in any data synthesis.	6
Summary measures	13	State the principal summary measures (e.g., risk ratio, difference in means).	6
Synthesis of results	14	Describe the methods of handling data and combining results of studies, if done, including measures of consistency (e.g., I^2) for each meta-analysis.	6/7

PRISMA 2009 Checklist

Section/topic	#	Checklist item	Reported on page #
Risk of bias across studies	15	Specify any assessment of risk of bias that may affect the cumulative evidence (e.g., publication bias, selective reporting within studies).	6
Additional analyses	16	Describe methods of additional analyses (e.g., sensitivity or subgroup analyses, meta-regression), if done, indicating which were pre-specified.	6/7
**RESULTS**			
Study selection	17	Give numbers of studies screened, assessed for eligibility, and included in the review, with reasons for exclusions at each stage, ideally with a flow diagram.	Figure 1
Study characteristics	18	For each study, present characteristics for which data were extracted (e.g., study size, PICOS, follow-up period) and provide the citations.	5
Risk of bias within studies	19	Present data on risk of bias of each study and, if available, any outcome level assessment (see item 12).	10
Results of individual studies	20	For all outcomes considered (benefits or harms), present, for each study: (a) simple summary data for each intervention group (b) effect estimates and confidence intervals, ideally with a forest plot.	Figures 2-7
Synthesis of results	21	Present results of each meta-analysis done, including confidence intervals and measures of consistency.	7-9
Risk of bias across studies	22	Present results of any assessment of risk of bias across studies (see Item 15).	10
Additional analysis	23	Give results of additional analyses, if done (e.g., sensitivity or subgroup analyses, meta-regression [see Item 16]).	9
**DISCUSSION**			
Summary of evidence	24	Summarize the main findings including the strength of evidence for each main outcome; consider their relevance to key groups (e.g., healthcare providers, users, and policy makers).	10-12
Limitations	25	Discuss limitations at study and outcome level (e.g., risk of bias), and at review-level (e.g., incomplete retrieval of identified research, reporting bias).	13
Conclusions	26	Provide a general interpretation of the results in the context of other evidence, and implications for future research.	14
**FUNDING**			
Funding	27	Describe sources of funding for the systematic review and other support (e.g., supply of data); role of funders for the systematic review.	NA

From: Moher D, Liberati A, Tetzlaff J, Altman DG, The PRISMA Group (2009). Preferred Reporting Items for Systematic Reviews and Meta-Analyses: The PRISMA Statement. PLoS Med 6(7): e1000097. doi:10.1371/journal.pmed1000097

For more information, visit: **www.prisma-statement.org**.
